# Sex-Based Differences in SGLT2i and GLP-1RA Use and Mortality in T2DM with Atherosclerotic Cardiovascular Disease

**DOI:** 10.3390/biomedicines14020404

**Published:** 2026-02-10

**Authors:** Hana Vaknin-Assa, Ammie Wolf, Ranin Hilu, Ela Giladi, Mustafa Gabarin, Ilya Losin, Yoav Arnson, David Pereg, Abid Assali, Ziad Arow

**Affiliations:** 1 Cardiology Department, Meir Medical Center, Kfar Saba 4428164, Israelaassali@clalit.org.il (A.A.); 2 Gray Faculty of Medical & Health Sciences, Tel-Aviv University, Tel-Aviv 6139001, Israel; 3 Internal Medicine C Department, Meir Medical Center, Kfar Saba 4428164, Israel

**Keywords:** sodium-glucose cotransporter-2 inhibitor (SGLT2i), glucagon-like peptide-1 receptor agonist (GLP-1RA), type 2 diabetes mellitus (T2DM), atherosclerotic cardiovascular disease (ASCVD)

## Abstract

**Background**: Sodium-glucose cotransporter-2 inhibitors (SGLT2i) and glucagon-like peptide-1 receptor agonists (GLP-1RA) remain underused in routine practice, particularly among women. **Aim**: This study evaluated gender differences in mortality among patients with T2DM and established atherosclerotic cardiovascular disease (ASCVD) and examined whether disparities in SGLT2i and GLP-1RA dispensing contribute to mortality. **Methods**: The CARDIAB cohort included 138,397 patients with T2DM and established ASCVD, categorized by gender into male and female groups. The primary endpoint was all-cause mortality, and the secondary outcome was the dispensing rates of SGLT2i and GLP-1RA. **Results**: Of the 138,397 patients, 40.3% were women and 59.7% were men. The overall dispensing rates of SGLT2i and GLP-1RA were 37.1% and 23.4%, respectively, and were significantly lower among women compared with men for both SGLT2i (27.8% vs. 43.3%; *p* < 0.001) and GLP-1RA (21.3% vs. 24.9%; *p* < 0.001). Women exhibited higher mortality rates, as reflected by deaths per 10,000 patient-years (9724 vs. 7744; *p* < 0.001). However, in multivariable analysis, gender was not an independent predictor of mortality. Notably, the use of cardioprotective medications was strongly associated with reduced mortality, with the greatest benefit observed for SGLT2i (HR 0.307; 95% CI 0.299–0.316; *p* < 0.001) and GLP-1RA (HR 0.466; 95% CI 0.451–0.482; *p* < 0.001). **Conclusions**: Women with T2DM and ASCVD were treated less often with SGLT2i and GLP-1RA, therapies strongly associated with lower mortality. Their higher unadjusted mortality appears to reflect undertreatment rather than sex-related risk. Action is needed to improve the use of these cardioprotective medications, especially in women.

## 1. Introduction

Type 2 diabetes mellitus (T2DM) combined with atherosclerotic cardiovascular disease (ASCVD) is associated with a substantially increased risk of cardiovascular mortality. Treatment with Sodium-glucose cotransporter 2 inhibitors (SGLT2i) and Glucagon-like peptide-1 receptor agonists (GLP-1RA) is associated with improved cardiovascular outcomes, including reduced mortality and morbidity, in this high-risk population [1,2,3]. Accordingly, leading international guidelines now provide Class I recommendations for SGLT2i and GLP-1RA in patients with T2D and elevated cardiovascular risk, independent of baseline glycemic control or HbA1c levels [4,5,6,7]. Despite well-established benefits and clear guideline recommendations, these treatments remain significantly underutilized in routine care and, particularly for SGLT2 inhibitors, this gap appears more pronounced among women [8,9,10,11]. The American Diabetes Association and the European Association for the Study of Diabetes also emphasize that women are underrepresented in cardiovascular outcome trials and receive less intensive management of cardiovascular risk factors, despite similar or greater benefit from these therapies compared to men [12]. The American Heart Association has likewise issued a call to action to address gender-based inequities in cardiovascular care and to promote equitable access to evidence-based therapies [13]. This study aims to examine the mortality rates by gender among patients with T2DM and ASCVD and to investigate the factors contributing to this disparity, with a particular emphasis on gender differences in the dispensing patterns of SGLT2i and GLP-1RA.

## 2. Material and Methods

### 2.1. Study Population

The CARdiovascular and DIABetes (CARDIAB) cohort study utilized the electronic health record database of Clalit Health Services (CHS). CHS is the largest integrated payer–provider healthcare organization in Israel, covering more than 4.5 million individuals, representing approximately 55% of the national population. The CARDIAB dataset is based on information from the Sharon-Shomron district, the second-largest district within CHS. Data retrieval was performed through the Clalit research data-sharing platform, supported by MDClone (MDClone Ltd., Be’er Sheva, Israel) (https://www.mdclone.com (accessed on 3 February 2026)).

The CARDIAB cohort comprised patients aged 18 years or older with T2DM and ASCVD during the period 2019–2024. ASCVD was defined as ischemic heart disease (IHD—myocardial infarction, percutaneous coronary intervention, or coronary artery bypass grafting), cerebrovascular accident (CVA) or transient ischemic attack (TIA), or peripheral vascular disease (PVD), defined as documented lower extremity arterial disease, prior peripheral revascularization (surgical or endovascular), or limb ischemia recorded in hospital or outpatient records. The index event was defined as either (1) the first clinical manifestation of ASCVD in individuals with pre-existing T2DM or (2) the initial diagnosis of T2DM in patients with established ASCVD. Patients with end-stage kidney disease receiving dialysis, as well as those who had been treated with SGLT2i or GLP-1RA before the index event, were excluded from the study. Collected data included patient demographics, clinical characteristics, diagnoses from both inpatient and outpatient settings, medical therapies, medication dispensing records, and laboratory results. All clinical diagnoses were identified using International Classification of Diseases, Ninth Revision (ICD-9) codes. Participants were stratified by gender (women vs. men). The primary endpoint was all-cause mortality following the index event. The secondary outcome was the dispensing rates of SGLT2i and GLP-1RA. Medication dispensing was defined as the provision of medication for a minimum duration of three months.

The study received approval from the local institutional ethics committee and was conducted in accordance with the Declaration of Helsinki. In accordance with Ministry of Health regulations, written informed consent was waived because the data were anonymized and retrospectively obtained from electronic medical records without direct patient contact.

### 2.2. Statistical Analysis

Descriptive Statistics and Group Comparisons: Baseline characteristics were described using standard descriptive statistics. Continuous variables were reported as mean ± standard deviation, while categorical variables were expressed as counts and percentages. Comparisons between groups were performed using appropriate statistical methods. Continuous variables were analyzed using the Wilcoxon rank-sum test. For categorical variables, Pearson’s chi-square test was applied when at least 80% of expected cell counts were ≥5; otherwise, Fisher’s exact test was used.

Incidence Analysis and Poisson Regression: Incidence rates were determined as the number of events divided by person-time at risk and reported per 10,000 person-years. Incidence rate ratios (IRRs) with corresponding 95% confidence intervals (CIs) were calculated using Poisson regression models with robust variance estimation to address potential overdispersion. Person-time of exposure was incorporated into the models as an offset term.

Time-to-Event Analysis: Kaplan–Meier survival curves were generated to assess event-free survival probabilities across study groups. Follow-up began at the index date and continued until the first occurrence of the cardiac outcome of interest, death, or the end of the follow-up period, whichever came first.

Cox Proportional Hazards Models: Cox proportional hazards regression analyses were performed to estimate hazard ratios (HRs) with corresponding 95% confidence intervals (CIs) for the relationship between study group and time to event. The proportional hazards assumption was evaluated using weighted residuals. Multivariable Cox models were developed with adjustment for pre-specified baseline covariates that were either clinically relevant or demonstrated imbalance between groups (standardized mean difference > 0.10) in univariable analyses. In addition to sex, the adjusted variables included age categories, cardiovascular risk factors, comorbid conditions, and other pertinent prognostic factors.

Statistical Software and Significance Threshold: All statistical analyses were conducted using R software (version 4.2.3; R Foundation for Statistical Computing, Vienna, Austria). A two-tailed *p*-value of less than 0.05 was considered to indicate statistical significance.

## 3. Results

### 3.1. Study Population Characteristics 

The study included 138,397 patients, of whom 40.3% (*n* = 55,828) were women (Table 1). The median follow-up duration was 65 months. Most patients were older than 60 years, with 41.7% aged 61–74 years and 43.4% aged ≥ 75 years; the overall mean age was 72.8 years. Women were significantly older than men (mean age 75 vs. 71 years, *p* < 0.0001). The proportion of patients aged ≥ 75 years was higher among women than men (53.7% vs. 36.4%), whereas men more frequently belonged to the intermediate age group of 61–74 years (45.4% vs. 36.2%, *p* < 0.001). Regarding cardiovascular risk factors prior to the index event, women had a higher prevalence of hypertension (92.4% vs. 87.3%, *p* < 0.0001) and obesity (70.8% vs. 57.0%, *p* < 0.0001), with higher mean BMI values (30.8 vs. 29.0 kg/m^2^, *p* < 0.0001). Men, in contrast, more frequently reported smoking (63.7% vs. 27.9%, *p* < 0.0001). The prevalence of dyslipidemia was similar between genders. With respect to additional comorbidities, women had higher rates of prior CVA/TIA (42.5% vs. 33.5%, *p* < 0.0001) and heart failure (HF) (29.1% vs. 27.8%, *p* < 0.0001), whereas men had higher prevalence of ischemic heart disease (78.1% vs. 64.1%, *p* < 0.0001), peripheral vascular disease (15.6% vs. 12.2%, *p* < 0.0001), and chronic kidney disease (CKD) (19.0% vs. 15.1%, *p* < 0.0001). No significant gender differences were observed in the proportion of patients belonging to the Arab minority in Israel or residing in peripheral geographic regions.

### 3.2. Cardioprotective Medication Dispensing Rates 

The overall dispensing rates of SGLT2i and GLP-1RA were 37.1% and 23.4%, respectively (Table 2). Dispensing rates were significantly lower among women compared with men for both SGLT2i (27.8% vs. 43.3%; *p* < 0.001) and GLP-1RA (21.3% vs. 24.9%; *p* < 0.001). In adjusted analyses, the gender difference remained statistically significant, with men more likely to receive SGLT2i (OR 1.790; 95% CI 1.743–1.839; *p* < 0.001) and GLP-1RA (OR 1.257; 95% CI 1.220–1.296; *p* < 0.001). Men had higher dispensing rates of all antiplatelet agents, including aspirin (93% vs. 91.5%), clopidogrel (47.8% vs. 34.8%), prasugrel (4.2% vs. 1.3%), and ticagrelor (5.6% vs. 2.8%). In contrast, women were more frequently dispensed beta-blockers (81.1% vs. 77.7%), angiotensin receptor blockers (ARBs) or angiotensin-converting enzyme inhibitors (ACEi) (88.1% vs. 86.3%), and Mineralocorticoid receptor antagonist (17.4% vs 15.3%). All these differences were statistically significant. No significant gender-based differences were observed in the dispensing of statins or other anti-diabetic medications.

### 3.3. Survival and Mortality Rates by Gender

**Figure 1:**
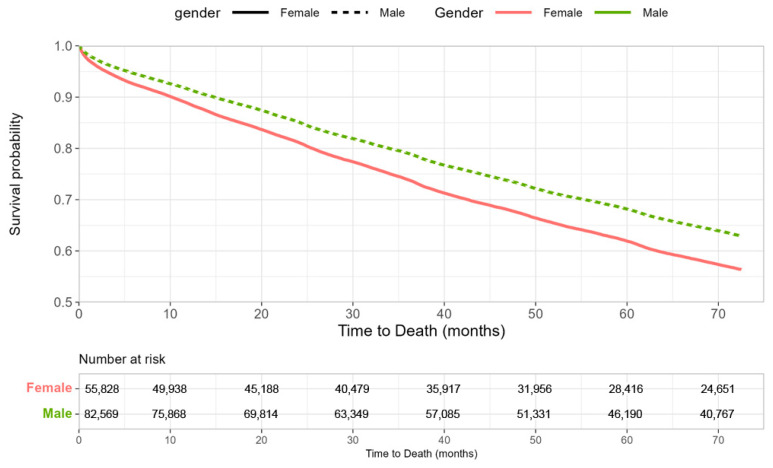
Kaplan–Meier Survival Curves by Gender.

**Figure 2:**
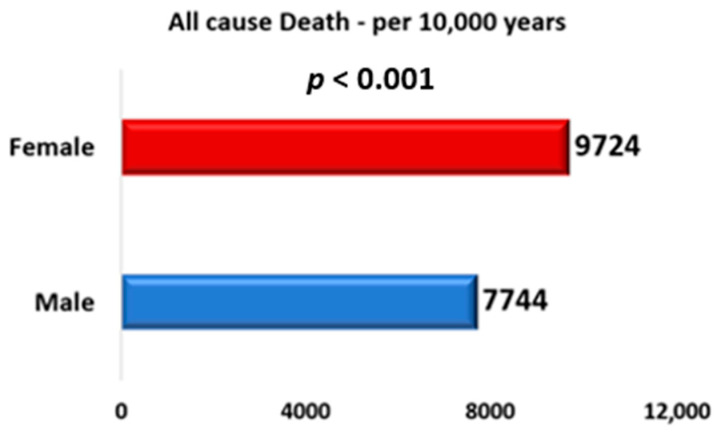
All cause death per 10,000 patient years.

Regarding the primary outcomes, Kaplan–Meier analysis showed lower survival among women (Figure 1), with higher mortality rates per 10,000 patient-years compared with men (9724 vs. 7744; *p* < 0.001) (Figure 2). However, in the multivariable analysis, gender was not an independent predictor of mortality (HR 1.009; 95% CI 0.989–1.030; *p* = 0.365). Instead, mortality risk was driven by advancing age and multiple baseline cardiovascular risk factors and comorbidities. Notably, the use of cardioprotective medications was associated with substantially lower mortality risk, with the strongest associations observed for SGLT2i (HR 0.307; 95% CI 0.299–0.316; *p* < 0.001) and GLP-1RA (HR 0.466; 95% CI 0.451–0.482; *p* < 0.001) (Table 3). To evaluate whether the association between cardioprotective therapy and mortality differed by sex, interaction terms between sex and treatment were added to the multivariable Cox model. The interaction between female sex and SGLT2 inhibitor use was not statistically significant (HR 0.947, 95% CI 0.887–1.011; *p* = 0.102). Similarly, the interaction between female sex and GLP-1 receptor agonist use was not significant (HR 0.938, 95% CI 0.857–1.027; *p* = 0.165).

## 4. Discussion

In this large real-world cohort of 138,397 patients with T2DM and established ASCVD, we found that, despite strong evidence supporting their cardiovascular benefits, the use of SGLT2i and GLP-1RA remained markedly low. Only 37.1% of patients received SGLT2i and 23.4% received GLP-1RA. Notably, substantial gender disparities were evident: women were significantly less likely than men to be treated with SGLT2i (27.8% vs. 43.3%) or GLP-1RA (21.3% vs. 24.9%). Regarding clinical outcomes, women exhibited lower unadjusted survival, with higher mortality rates per 10,000 patient-years compared with men (9724 vs. 7744). However, after adjustment for age and comorbidities, gender was not an independent predictor of mortality. Instead, mortality risk was driven by age and baseline cardiovascular risk factors. Importantly, cardioprotective treatments were strongly associated with reduced mortality, with SGLT2i (HR 0.307; 95% CI 0.299–0.316) and GLP-1RA (HR 0.466; 95% CI 0.451–0.482) showing the most substantial survival benefit. This suggests that the observed gender disparity in mortality is driven by suboptimal treatment among women. Importantly, we found no significant interaction between sex and treatment effect for either SGLT2i or GLP-1RA, suggesting that the survival benefit associated with these therapies is similar in women and men. This supports the concept that the observed sex disparity in outcomes is more likely related to differences in treatment exposure rather than differences in therapeutic effectiveness. It is noteworthy that the gap in dispensing rates between men and women was smaller for GLP-1RA compared to SGLT2i, which may be attributed to the higher prevalence of obesity, higher BMI, and history of CVA/TIA among women in this cohort.

Several studies have examined the undertreatment of women with cardioprotective therapies. Eberly et al. [8] evaluated SGLT2i treatment rates among patients with T2DM and coexisting HF, CKD, or ASCVD and found that only 8.7% received an SGLT2i during the study period. Female sex, Black race, and lower socioeconomic status were all independently associated with lower SGLT2i prescription rates. Another study evaluated patients with T2DM and HF and found that only 17.3% received an SGLT2 inhibitor. Women had significantly lower SGLT2i prescription rates compared with men, leading the authors to conclude that greater awareness and implementation of guideline-directed therapy are urgently needed, particularly for female HF patients [10]. Dixon et al. [11] evaluated SGLT2i and GLP-1RA prescription patterns in more than 22,000 patients with T2DM and ASCVD, HF, or CKD, and found that only 17% of eligible patients received these therapies. Funck et al. [14] analyzed more than 70,000 patients with new-onset T2DM and ASCVD and found that female gender was associated with lower initiation rates of SGLT2i and GLP-1RA. Finally, the 2022 American Heart Association call to action for cardiovascular Disease in women [13] highlights persistent sex-based disparities in cardiovascular risk, awareness, access to care, and delivery of evidence-based treatments. The advisory emphasizes that women remain underdiagnosed, undertreated, and disproportionately affected by social determinants of health. Our study adds important real-world evidence to this growing body of literature by demonstrating not only that women receive SGLT2i and GLP-1RA at lower rates, but also that this undertreatment is associated with increased mortality among women.

These findings highlight a critical gap in the implementation of evidence-based cardioprotective therapies among patients with T2DM and ASCVD, particularly among women. The significantly lower use of SGLT2i and GLP-1RA in women, despite their strong association with reduced mortality, suggests substantial missed opportunities for cardiovascular risk and mortality reduction. Improving equitable access to and uptake of these therapies may meaningfully reduce mortality disparities and enhance outcomes in routine clinical practice. These results underscore the urgent need for healthcare systems and policymakers to implement strategies that promote broader use of these therapies in both genders, with particular emphasis on women, a historically undertreated group compared with men [15,16,17].

The magnitude of mortality reduction associated with SGLT2i use in this real-world cohort appears greater than that reported in randomized cardiovascular outcome trials [1,18,19]. This discrepancy likely reflects fundamental differences between observational effectiveness studies and randomized controlled trials. In routine clinical practice, treatment allocation is not random and may preferentially occur in patients who are more clinically stable, more adherent to care, or more closely followed, introducing healthy-user, survivor, and selection biases. In addition, medication exposure in our study was based on dispensing records rather than protocol-driven initiation, and residual confounding despite multivariable adjustment cannot be excluded. Therefore, the observed associations may partly reflect differences in patient selection and healthcare engagement rather than a purely biological treatment effect.

This study has several limitations. First, as a retrospective observational analysis, it is subject to residual confounding and healthy-user or healthy-treatment bias, despite adjustment for multiple covariates. Second, the database did not include details on specific causes of death, restricting the analysis to all-cause mortality as the main endpoint. Third, treatment with GLP-1RA or SGLT2i was identified as a minimum three dispensations; however, information regarding actual treatment duration, persistence, adherence, timing of initiation from the index event, percentage of days covered, and possible simultaneous use of both agents was lacking. Because treatment exposure was assessed after the index event and modeled as a fixed variable, we cannot exclude survivor bias or confounding related to patient clinical stability at the time of treatment initiation. Fourth, medication dispensing data do not guarantee clinical adherence, which may have influenced outcome estimates. In addition, the database did not consistently capture diabetes duration, which is an important prognostic factor and may have influenced survival outcomes. Finally, although the CARDIAB cohort represents a substantial national real-world population, the applicability of these results to different healthcare systems or countries should be approached with caution.

## 5. Conclusions

Women with T2DM and ASCVD were treated less often with SGLT2i and GLP-1RA, therapies strongly linked to lower mortality. Their higher unadjusted mortality reflects undertreatment rather than sex-related risk. Improving equitable use of these medications, especially among women, is essential to reducing preventable deaths in this population.

## Figures and Tables

**Table 1:** Baseline characteristics according to treatment group.

Treatment Group	Overall	Female	Male	*p*-Value
n	138,397	55,828 (40.3%)	82,569 (59.7%)	
Age, years (mean ± SD)	72 ± 11	75 ± 11	71 ± 11	<0.001
Age Group, n (%)				<0.001
19–60	20,632 (14.9)	5589 (10)	15,043 (18.2)	
61–74	57,738 (41.7)	20,233 (36.2)	37,505 (45.4)	
75+	60,027 (43.4)	30,006 (53.7)	30,021 (36.4)	
Sector, n (%)				0.060
Arab	24,035 (17.4)	9560 (17.1)	14,475 (17.5)	
General population	114,362 (82.6)	46,268 (82.9)	68,094 (82.5)	
Periphery residence, n (%)	7138 (5.2)	2844 (5.1)	4294 (5.2)	0.380
Cardiovascular risk factors:				
Obesity, n (%)	86,583 (62.6)	39,527 (70.8)	47,056 (57)	<0.001
BMI (mean ± SD)	29.7 ± 5.5	30.8 ± 6.2	29.0 ± 4.8	<0.001
Dyslipidemia, n (%)	97,351 (70.3)	39,108 (70.1)	58,243 (70.5)	0.051
Hypertension, n (%)	123,633 (89.3)	51,562 (92.4)	72,071 (87.3)	<0.001
CKD, n (%)	24,123 (17.4)	8409 (15.1)	15,714 (19.0)	<0.001
Smokers, n (%)	67,823 (49.3)	15,448 (27.9)	52,375 (63.7)	<0.001
Cardiovascular disease:				
IHD, n (%)	100,328 (72.5)	35,813 (64.1)	64,515 (78.1)	<0.001
CVA/TIA, n (%)	51,329 (37.1)	73,702 (42.5)	27,627 (33.5)	<0.001
PVD, n (%)	19,665 (14.2)	6.795 (12.2)	12,870 (15.6)	<0.001
Heart Failure, n (%)	39,179 (28.3)	16,230 (29.1)	22,949 (27.8)	<0.001
Laboratory Parameters				
LDL-C, mg/dL (mean ± SD)	95 ± 35	97 ± 35	94 ± 35	<0.001
HDL-C, mg/dL (mean ± SD)	44 ± 11	49 ± 12	41 ± 10	<0.001
Total Cholesterol, mg/dL (mean ± SD)	173 ± 45	179 ± 44	169 ± 45	<0.001
Triglycerides, mg/dL, (mean ± SD)	166 ± 122	160 ± 96	170 ± 137	<0.001

BMI = Body mass index; CKD = Chronic kidney disease; IHD = Ischemic heart disease; CVA = Cerebrovascular accident; TIA = Transient ischemic attack; PVD = Peripheral vascular disease.

**Table 2:** Dispensing rates of cardioprotective medications by gender.

Treatment Group	Overall	Female	Male	*p*-Value
n	138,397	55,828	82,569	
SGLT2i and GLP-1RA				
SGLT2i, n (%)	51,279 (37.1)	15,509 (27.8)	35,770 (43.3)	<0.001
GLP-1RA, n (%)	32,404 (23.4)	11,874 (21.3)	20,530 (24.9)	<0.001
Other Medications:				
Statins, n (%)	131,150 (94.8)	52,878 (94.7)	78,272 (94.8)	0.512
Aspirin, n (%)	127,855 (92.4)	51,056 (91.5)	76,799 (93)	<0.001
Clopidogrel, n (%)	58,931 (42.6)	19,436 (34.8)	39,495 (47.8)	<0.001
Prasugrel, n (%)	4233 (3.1)	730 (1.3)	3503 (4.2)	<0.001
Ticagrelor, n (%)	6145 (4.4)	1550 (2.8)	4595 (5.6)	<0.001
Ezetimibe, n (%)	15,071 (10.9)	5852 (10.5)	9219 (11.2)	<0.001
ACEi/ARB, n (%)	120,414 (87.0)	49,170 (88.1)	71,244 (86.3)	<0.001
Beta-blockers, n (%)	109,432 (79.1)	45,277 (81.1)	64,155 (77.7)	<0.001
MRA, n (%)	22,355 (16.2)	9705 (17.4)	12,650 (15.3)	<0.001
Other anti-diabetes drugs *, n (%)	122,056 (88.2)	49,288 (88.3)	72,768 (88.1)	0.379

SGLT2i = Sodium-glucose cotransporter 2 inhibitor; GLP-1RA = Glucagon-like peptide-1 receptor agonist; ACEi = Angiotensin converting enzyme inhibitor; ARB = Angiotensin receptor blocker; MRA = Mineralocorticoid receptor antagonist. * Including Biguanides, DPP-4 inhibitors, Sulfonylureas, Meglitinides, Thiazolidinediones, Alpha-glucosidase inhibitors, and Insulin therapy.

**Table 3:** Multivariable Cox Proportional Hazards Model for Mortality Stratified by Gender *.

Characteristic	HR ^1^	95% CI ^1^	*p*-Value
gender			
Female		—	
Male	1.009	0.989, 1.030	0.3650
Age Group			
19–60		—	
61–74	1.694	1.622, 1.768	<0.001
75+	3.284	3.147, 3.427	<0.001
Smoking	1.100	1.079, 1.122	<0.001
Hypertension	1.160	1.113, 1.210	<0.001
CVA/TIA	1.331	1.305, 1.358	<0.001
Chronic Kidney Disease	1.593	1.560, 1.626	<0.001
PVD	1.349	1.318, 1.380	<0.001
GLP-1RA	0.466	0.451, 0.482	<0.001
SGLT2i	0.307	0.299, 0.316	<0.001
ACEi/ARB	0.969	0.951, 0.987	0.009
Statins	0.827	0.792, 0.864	<0.001
Ezecor	0.843	0.817, 0.870	<0.001
Aspirin	0.895	0.861, 0.931	<0.001
Effient	0.851	0.792, 0.914	<0.001
Brilinta	0.867	0.825, 0.911	<0.001

CVA = Cerebrovascular accident; TIA = Transient ischemic attack; PVD = Peripheral vascular disease; SGLT2i = Sodium-glucose cotransporter 2 inhibitor; GLP-1RA = Glucagon-like peptide-1 receptor agonist; ACEi = Angiotensin converting enzyme inhibitor; ARB = Angiotensin receptor blocker; ^1^ HR = Hazard Ratio, CI = Confidence Interval; * Only parameters that remained statistically significant in the multivariable Cox proportional hazards model are presented in this table.

## Data Availability

The data underlying this article will be shared on reasonable request to the corresponding author.

## Abbreviations and Acronyms

SGLT2i	Sodium-glucose cotransporter-2 inhibitor
GLP-1RA	Glucagon-like peptide-1 receptor agonist
T2DM	Type 2 diabetes mellitus
ASCVD	Atherosclerotic cardiovascular disease
HF	Heart failure
CVA	Cerebrovascular accident
TIA	Transient ischemic attack
